# PsychoPy2: Experiments in behavior made easy

**DOI:** 10.3758/s13428-018-01193-y

**Published:** 2019-02-07

**Authors:** Jonathan Peirce, Jeremy R. Gray, Sol Simpson, Michael MacAskill, Richard Höchenberger, Hiroyuki Sogo, Erik Kastman, Jonas Kristoffer Lindeløv

**Affiliations:** 1 School of Psychology, University of Nottingham, Nottingham, UK; 2 Knack, Inc., Okemos, MI USA; 3 iSolver Software Solutions, Osgoode, Ontario Canada; 4 Department of Medicine, University of Otago, Christchurch, New Zealand; 5 New Zealand Brain Research Institute, Christchurch, New Zealand; 6 Cognitive Neuroscience, Institute of Neuroscience and Medicine (INM-3), Research Center Jülich, Jülich, Germany; 7 Faculty of Law and Letters, Ehime University, Matsuyama, Ehime Japan; 8 Department of Psychology and Center for Brain Science, Harvard University, Cambridge, MA USA; 9 CCN, Department of Psychology and Communication, Aalborg University, Aalborg, Denmark

**Keywords:** Psychology, Software, Experiment, Open-source, Open science, Reaction time, Timing

## Abstract

PsychoPy is an application for the creation of experiments in behavioral science (psychology, neuroscience, linguistics, etc.) with precise spatial control and timing of stimuli. It now provides a choice of interface; users can write scripts in Python if they choose, while those who prefer to construct experiments graphically can use the new Builder interface. Here we describe the features that have been added over the last 10 years of its development. The most notable addition has been that Builder interface, allowing users to create studies with minimal or no programming, while also allowing the insertion of Python code for maximal flexibility. We also present some of the other new features, including further stimulus options, asynchronous time-stamped hardware polling, and better support for open science and reproducibility. Tens of thousands of users now launch PsychoPy every month, and more than 90 people have contributed to the code. We discuss the current state of the project, as well as plans for the future.

Computers are an incredibly useful, almost ubiquitous, feature of the modern behavioral research laboratory, freeing many scientists from the world of tachistoscopes and electrical engineering. Scientists have a large range of choices available, in terms of hardware (e.g., mouse vs. touchscreen) and operating system (Mac, Windows, Linux, or mobile or online platforms), and they no longer need to have a degree in computer science to make their experiment run with frame-by-frame control of the monitor.

A wide range of software options are also available for running experiments and collecting data, catering for various needs. There are commercial products, such as E-Prime (Psychology Software Tools Inc., Sharpsburg, PA, USA), Presentation (Neurobehavioral Systems Inc., Berkeley, California, USA), Experiment Builder (SR Research Ltd., Canada), and Psykinematrix (Kybervision, LLC, Japan). A relatively new possibility, however, has been the option to use free open-source products, provided directly by academics writing tools for their own labs and then making them freely available to others.

The most widely used example, to date, began as a set of C routines, called VideoToolbox, written by Denis Pelli, initially to carry out studies in vision science (Pelli, 1997). David Brainard wrote MATLAB wrappers around the VideoToolbox library, with some additional pure MATLAB code, and called the package *Psychophysics Toolbox* (Brainard, 1997). This has now gone through several iterations and substantial rewriting, especially by Mario Kleiner in the most recent version, Psychtoolbox 3 (Kleiner, Brainard, & Pelli, 2007). Psychtoolbox shows how successful these projects can be. After 20 years, it is still in active development and has been used extensively in research. It also shows how popular the open-source movement has become; in 2004, the Psychophysics Toolbox article (Brainard, 1997) received 123 citations (according to Google Scholar), whereas in 2018 it received 1,570.

Open-source packages have several attractive features beyond being free. Having access to all the source code means that a scientist can examine what is happening “under the hood” and can extend or adapt the code themselves if the package does not already have the features or performance they need. Most open-source packages are written in high-level interpreted languages, typically MATLAB or Python. This has made it relatively easy to provide support for all platforms, so the scientist can develop and run the same study on any machine. Most important to many people, however, is the principle of openness and the sense that this is good practice for replicable research.

In terms of the choice of available scripting languages, while there are again many options (e.g., R, MATLAB, Mathematica, or Java), Python is one of the most popular languages in the world at the time of writing. The PopularitY of Programming Language project (PYPL) has analyzed Google searches for programming tutorials and found that over 25% of searches are for Python tutorials, as compared with 2.5% for MATLAB and 4% for R (see http://pypl.github.io/PYPL.html for up-to-date statistics). Python is so useful as a scripting language that MacOS and many flavors of Linux provide it as standard in their operating systems. That popularity means that the language receives a great deal of support from hardware manufacturers and programmers from all spheres.

The PsychoPy project began in 2002, as a Python library to conduct visual neuroscience experiments in Jonathan Peirce’s lab. It developed a small following of Python enthusiasts in the field, and gradually it grew to provide further stimuli and features (Peirce, 2007, 2009). At that point, PsychoPy provided a useful set of stimuli and methods and a basic editor with which to write code, but it required users to program their experiments, which made it inaccessible to nonprogrammers, including most undergraduate psychology students.

The question was how to enable nonprogrammers to use PsychoPy. Ideally, the package should be accessible enough for typical undergraduates in psychology (who are often quite averse to programming), while also offering the flexibility required for professional researchers to build a range of precise experiments.

This led to the addition of a graphical experiment creation interface called the Builder, the defining feature in the development of PsychoPy2. In addition to the Builder, which freed users from the need to be computer programmers, a large number of improvements and new features have been added. Additionally, PsychoPy has adopted a more robust development and testing workflow and has benefited from the growth of a supportive online community. With the bulk of that phase of development now complete—the Builder interface has become a relatively stable tool and has shown itself capable of running a wide range of studies—this article provides a brief summary of the features and changes that have come about over the last 10 years of development of PsychoPy.

It is beyond the scope of this article to teach readers how to *use* the software. For that there are numerous didactic resources available, such as YouTube videos (e.g., https://www.youtube.com/playlist?list=PLFB5A1BE51964D587), the demo menus that are built into the application, the extensive online documentation at http://www.psychopy.org, and even a textbook (Peirce & MacAskill, 2018).

## Other packages

At the time that the core PsychoPy library was written, the other comparable packages were Vision Egg (Straw, 2008) and PyEPL (Geller, Schlefer, Sederberg, Jacobs, & Kahana, 2007), both of which subsequently ceased development. Since 2008, numerous additional libraries have been created in Python, such as Expyriment (Krause & Lindemann, 2014), PyGaze (Dalmaijer, Mathôt, & Van der Stigchel, 2014), mPsy (https://wisions.github.io/mPsy/), and SMILE (http://smile-docs.readthedocs.io/). In comparison to these, PsychoPy offers a broader list of stimulus options, experimental designs, response options (such as rating scales), and hardware support, as well as a larger community of active developers.

Most critically, however, the other libraries do not offer a graphical interface to create studies, which limits their suitability for undergraduate teaching. Another Python-based application, OpenSesame (Mathôt, Schreij, & Theeuwes, 2012), was, however, developed around the same time as the PsychoPy Builder interface. PsychoPy and OpenSesame remain, to our knowledge, the most versatile open-source experiment-building packages currently available, and we compare them in the following section. There was also an open-source Macintosh application called PsyScopeX (http://psy.ck.sissa.it/ ), buCupdate since 2015.

## Builder

**Fig. 1:**
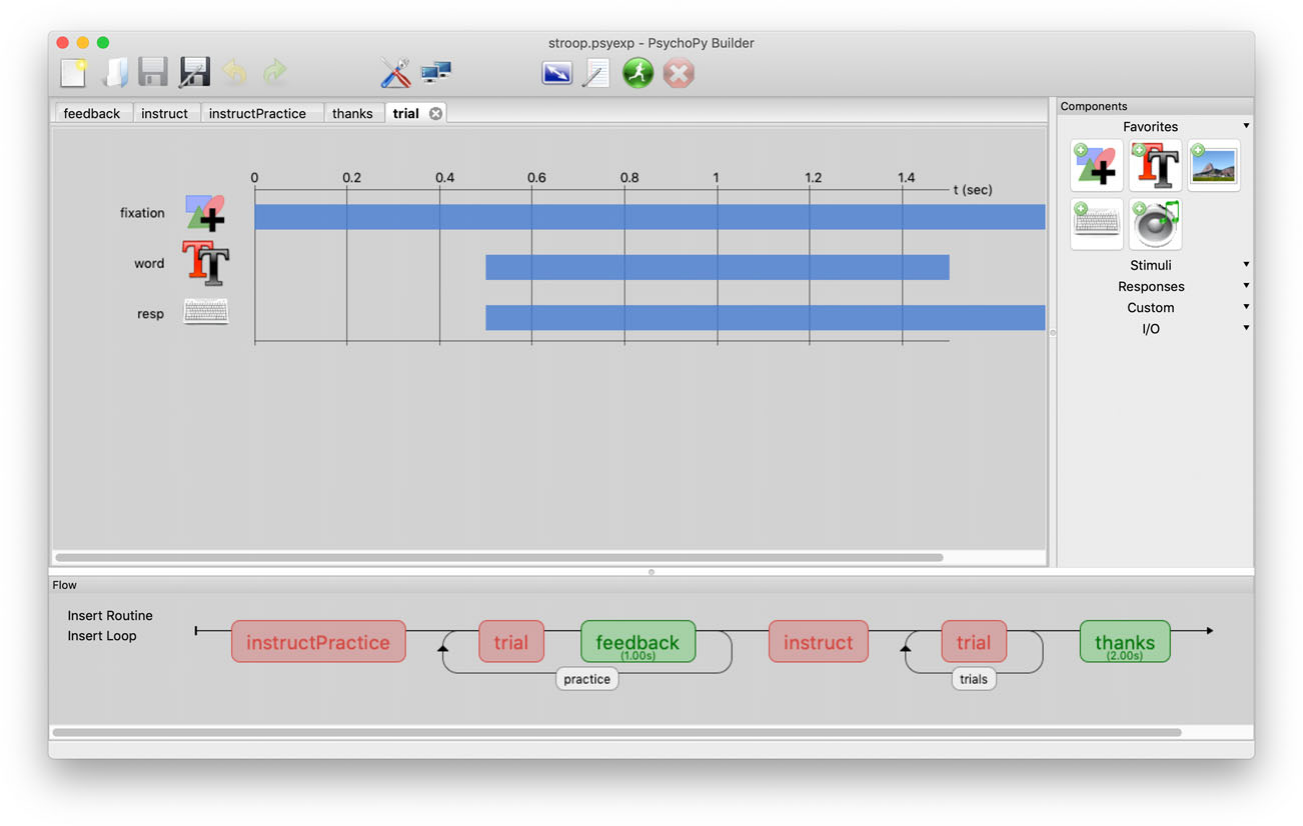
The PsychoPy Builder interface. The right-hand panel contains the Components that can be added to the experiment, organized by categories that can be expanded or collapsed. These Components can be added into Routines and appear like “tracks” in the Routine panel. In the demo shown here, in the Routine named “trial,” we simply present a word after a 500 ms pause and simultaneously start monitoring the keyboard for responses, but any number of Components can be set to start and stop in a synchronous or asynchronous fashion. The bottom panel of the interface shows the Flow of the experiment: the sequence in which the Routines will be presented, including the occurrence of any Loops in which we can repeat trials and/or blocks and control the randomization of conditions. Users report that this view is a highly intuitive and flexible way to implement their experimental designs

The idea of the Builder interface was to allow the user to create a graphical representation of an experiment. From this, the software would then generate a Python script to actually run the experiment. We wanted something that would be cross-platform, open and free, and that would support Python programming when experiments needed extending. We also wanted to provide stimuli that were dynamic, with stimulus attributes that could be updated on each screen refresh as specified directly from the graphical interface, which was not possible (or certainly was not easy) using other graphical interfaces An image of the Builder interface can be seen in Fig. 1.

### How does Builder work?

In PsychoPy Builder, an experiment is described by a set of *Routines*, which contain a set of one or more *Components*, such as stimuli and response options. The Components in the Routines can be thought of as a series of tracks in a video- or music-editing suite; they can be controlled independently in time—that is, onsets and offsets—but also in terms of their properties. The last part of the experiment description is the *Flow*: a flow diagram that controls how the Routines relate to each other. It contains the Routines themselves, as well as *Loops* (which repeat the Routines they encompass). The Flow has no “knowledge” of time per se; it simply runs each Routine immediately after the previous one has ended. The experimental timing is controlled by specifying the times of onset and offset of the stimuli and of response-gathering events within the Routines themselves.

This experiment description is internally stored in terms of standard Python objects: a Python list of Routines, each of which is a list of Components, which are themselves essentially a Python dictionary of parameters and, finally, a list of items on the Flow. Builder saves the experiment as standard XML-formatted text files using the open-standard *psyexp* format (read more at http://www.psychopy.org/psyexp.html). These files need not be specific to PsychoPy or Python; any system that can interpret a simple XML file could theoretically receive a Builder-generated experiment file and use that description to conduct the study, if it has a similar set of stimulus features.

The first job of the Builder interface is to provide a graphical means to create and represent these *psyexp* experiment descriptions. The second job is to be able to generate, from those descriptions, working code to actually run the experiments. This step is made relatively easy by Python’s powerful text-handling structures and object-oriented syntax. Users can compile and inspect the resulting script at the click of a button.

In general, that output script is a Python/PsychoPy script, but the interface could output scripts for alternative targets as well. Since the Builder is merely generating text, representing code based on the Components and Flow, it is only a matter of developer resources to expand this capability in order to generate experiments written in languages other than Python—for example, to generate a Psychophysics Toolbox script (Brainard, 1997; Kleiner et al., 2007; Pelli, 1997) in the MATLAB language. Indeed, we are now working on an HTML/JavaScript output so that Builder experiments can also run in a web browser (which is not possible with Python code).

The generated Python code is well-formatted and heavily commented, to allow users to learn more about Python programming—and the PsychoPy package in particular—in a top-down fashion. This allows the user to adapt that output script and then run the adapted version themselves, although this is a one-way road—scripts cannot be converted back into the graphical representation.

Additionally, the Builder also provides a *Code Component* that allows users to execute arbitrary Python code at any of the same points available to standard Components (at the beginning of the experiment, beginning of a trial, every screen refresh, etc.). These Code Components allow the experimenter to add a high level of customization to the study without leaving the comfort of the Builder interface. This provides a balance between ease of use (via the graphical interface) and flexibility (allowing out-of-the-ordinary requirements to be implemented in custom code).

OpenSesame (Mathôt et al., 2012) has a similar goal of providing a graphical interface for specifying experiments. OpenSesame uses, among several options, the PsychoPy Python module as a back end to present stimuli and has become popular for its ease of use and slick interface. It differs from PsychoPy mainly in that (1) OpenSesame represents the flow in a nested list, similar to E-Prime, where PsychoPy has a horizontal flow with loops; (2) in Routines, the PsychoPy interface emphasizes the temporal sequence of components, where the Open Sesame interface emphasizes their spatial layout; (3) PsychoPy allows the experimental and stimulus parameters to vary dynamically on every screen refresh during a routine, whereas OpenSesame requires stimuli to be pregenerated; and (4) PsychoPy generates Python scripts that can be exported and run independently of the graphical interface.

### How well does the Builder achieve its goals?

#### Intuitive enough for teaching

The aim was to allow nonprogrammers, including undergraduates, to be able to generate experiments. The best way for the reader to judge whether this aim has been achieved is perhaps to watch one of the walk-through tutorials on YouTube (e.g., https://www.youtube.com/playlist?list=PLFB5A1BE51964D587). The first of these videos shows, in 15 min and assuming no prior knowledge, how to create a study, run it, and analyze the generated data.

PsychoPy’s Builder interface is being used for undergraduate teaching in many institutions, allowing students to create their own experiments. At the School of Psychology, University of Nottingham, we previously used E-Prime for our undergraduate practical classes. In the academic year September 2010–2011, our first-year undergraduates spent the first half using PsychoPy and the second using E-Prime. We surveyed the students at the end of that year and, of the 60 respondents, 31 preferred PsychoPy to E-Prime (as compared with nine preferring E-Prime, and the remainder expressing no preference), and 52 reported that they could “maybe,” “probably,” or “definitely” create a study on their own following the five sessions with PsychoPy. PsychoPy has gained a number of usability improvements since then, and Nottingham now uses PsychoPy for all its undergraduate classes.

#### Flexible enough for high-quality experiments

**Fig. 2:**
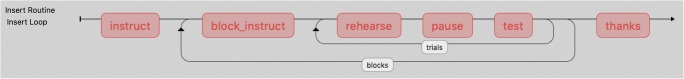
A more complex Flow arrangement. Loops and Routines can be nested in arbitrarily complex ways. PsychoPy itself is agnostic about whether a Loop designates trials, a sequence of stimuli *within* a trial, or a sequence of blocks *around* a loop of trials, as above. Furthermore, the mechanism for each loop is independent; it might be sequential, random, or a something more complex, such as an interleaved staircase of trials

We aimed to generate software that can implement most “standard” experiments to satisfactory levels of temporal, spatial, and chromatic accuracy and precision. In terms of features, the Builder can make use nearly all the stimuli in the PsychoPy library with no additional code. For instance, it can present images, text, movies, sounds, shapes, gratings (including second-order gratings), and random-dot kinematograms. All of these stimuli can be presented through apertures, combined with alpha-blending (transparency), and updated in most of their parameters on every screen refresh. Builder also supports inputs via keyboard, mouse, rating scales, microphone, various button boxes, and serial and parallel ports. It also supports a wide range of experiment structures, including advanced options such as interleaved staircase (e.g., QUEST) procedures. The use of arbitrary loop insertions, which can be nested and can be inserted around multiple other objects, allows the user to create a wide range of experimental flows. Figure 2 is a screenshot of one such Builder representation of an experimental flow.

At times, an experimenter will require access to features in the PsychoPy library that have not been provided directly as part of the graphical interface (often to keep the interface simple), or will want to call external Python modules beyond the PsychoPy library itself. This can be achieved by inserting snippets of custom code within a Code Component, as described above.

As evidence that PsychoPy is used by professional researchers, and not just as a teaching tool, according to Google Scholar, the original article describing PsychoPy (Peirce, 2007) now has *over 1,800 citations*. Most of these are empirical studies in which the software was used for stimulus presentation and response collection. The Builder interface is not only used by nonprogrammers, but also by researchers perfectly adept at programming, who find that they can create high-precision studies with greater efficiency and fewer errors by using this form of “graphical programming.”

**Fig. 3:**
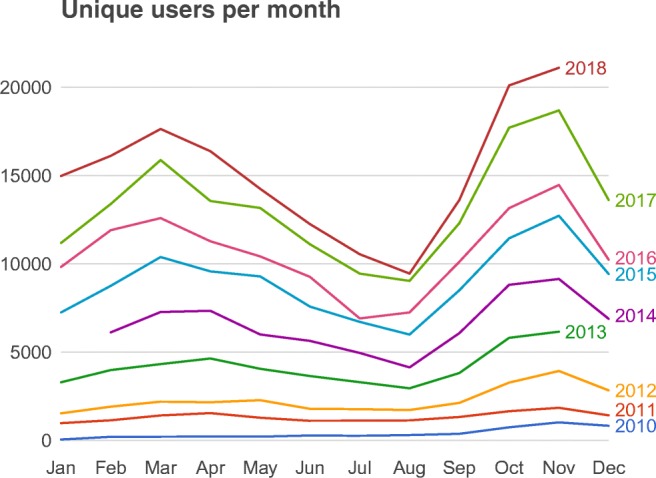
Users per month, based on unique IP addresses launching the application. These figures are underestimates, due mostly to the fact that multiple computers on a local area network typically have a single IP address. We can also see the holiday patterns of users, with dips in usage during Christmas and the Northern hemisphere summer

Indeed, several of the authors of this article use the Builder interface rather than handwritten Python code, despite being very comfortable with programming in Python. Overall, the clearest indication that people find PsychoPy both easy to use and flexible is the growth in user numbers since the Builder interface was first released (see Fig. 3). We have seen user numbers grow from a few hundred regular users in 2009 to tens of thousands of users per month in 2018.

#### Precision and accuracy

The Builder interface includes provision for high-precision stimulus delivery, just as with the code-driven experiments. Notably, the user can specify stimulus durations in terms of number of frames, for precise short-interval timing. PsychoPy will handle a range of issues, such as ensuring that trigger pulses to the parallel port are synchronized to the screen refresh. Builder-generated scripts are oriented around a drawing and event loop that is synchronized to the regular refresh cycle of the computer monitor. Hence, in general, the presentation of visual stimuli is both temporally accurate (being presented at the desired time and for the desired duration) and precise (with little variability in those times). One published study suggested that the temporal precision of PsychoPy’s visual stimuli was poor (Garaizar, Vadillo, López-de-Ipiña, & Matute, 2014), but this was an artifactual finding due to the authors using a prototype version of the Builder interface (v1.64, from 2011, which did carry an explicit warning that it should not be used for precision studies). The authors subsequently reran their analysis, using an official production-ready release (v1.80, 2014). Using the timing approach recommended in the documentation, they found very good timing of visual stimulus display, for “normal usage” (Garaizar & Vadillo, 2014).

A current limitation of temporal stimulus accuracy and precision, however, is the presentation of sound stimuli. There can be a lag (i.e., impaired accuracy) of sound onset, potentially up to tens of milliseconds, with associated trial-to-trial variability in those times of onset. Sound presentation relies on one of several underlying third-party sound libraries, and performance can vary across operating systems and sound hardware. The authors are currently conducting objective testing of performance across all these factors and updating PsychoPy’s sound library to one with better performance.

## Features and enhancements

As well as providing this new interface, making it easier for researchers at all levels to create experiments, there have been a large number of new features added to the PsychoPy Python library since the time of the last publication about the package (Peirce, 2009). Most notably, (1) researchers have the option to choose which version of PsychoPy to run the experiment on; (2) the range of stimuli that can be generated “out of the box” has grown considerably, as have the options for manipulating the existing stimulus types; and (3) increased support is available for external hardware and asynchronous response inputs.

### Choosing the software version at run time

One issue for reproducible and open science is that software releases do not always maintain compatibility from one version to another, and changes to software may have very subtle effects on stimulus presentation, response collection, and experimental flow. One unattractive solution is that users retain the same version of the software in the lab and avoid upgrading. This precludes users (and their colleagues) from accessing new features and benefiting from important bug fixes. To circumvent such issues, PsychoPy now allows the user to specify which version of the library to use for running the experiment, regardless of which version is currently installed. Typically, this will be the PsychoPy version in which the experiment was initially created. This can be done in the Experiment Settings of the Builder interface, or in code via the useVersion() function (see the top of Code Snippet 1). The specified version will be used to interpret the script, regardless of what PsychoPy version is currently installed.

The idea is that the user should get the experiment working correctly in the current latest version of the software and test it thoroughly in that version. For instance, experimenters should ensure that data files contain the necessary values by actually performing an analysis. They should ensure that the timing is correct, preferably with a Black Box Toolkit (Plant & Quinlan, 2013) or similar hardware. When they are confident that the study runs as intended, they should then “freeze” the experiment so that it will continue to use that version of the PsychoPy library indefinitely, even when the lab version of the PsychoPy application is itself updated. This is optional: Users who do want the latest features, and do not mind occasionally updating their code when PsychoPy necessarily introduces incompatible changes, can simply ignore the useVersion setting.

Even if the script requests a version from the “future” (i.e., one that has never actually been installed locally), PsychoPy will fetch it online as needed. If the experimental script does *not* explicitly specify a version, it will simply run using the latest *installed* version. Hence, this capability ensures both backward *and* forward capability.

We should note that there are still limitations to this system when the version being requested is not compatible with the Python installation or dependencies. The user cannot, for instance, request version 1.84.0 using an installation of Python 3, because compatibility with that version of Python was only added in PsychoPy 1.90.0.

### New stimuli and added features

#### Rating scales

PsychoPy now provides rating scales, in both its Python library and as a Component in the Builder interface. Ratings can be collected in a range of ways, from standard Likert-style scales to scales with a range of gradations, or continuous “visual analog” scales. These are highly customizable objects that allow many aspects to be controlled, including the text of a confirmation button, the shape and style of the response slider, and independent colors of various parts of the scale.

#### Movies

Movie stimuli were already available in 2008, but their reliability and efficiency have improved. Movies remain a technically challenging stimulus, but the recent improvements in performance mean that a fast computer running a recent version of PsychoPy should be able to present high-definition video smoothly.

#### Element arrays

The *ElementArrayStim* is a stimulus allowing the display of a large array of related elements in a highly optimized fashion. The key optimization is that the code can modify an entire array of objects in one go, leveraging the power of the graphics card to do so. The only constraint is that each element must use the same texture (e.g., a grating or an image) and mask, but the elements can differ in almost every other possible way (e.g., each having its own color, position, size, opacity, or phase). Hundreds or thousands of objects can be rendered by this means (in tasks such as visual search arrays or global form patterns), or instead as an array of simple masks that can gradually be *removed*. Currently, this stimulus is only available using code (either in scripts or as Code Components in the Builder interface), because it is inherently an object that needs programmatic control. See Code Snippet 1 for an example.

#### Geometric shapes

Users can now create vector-based shapes by specifying points geometrically, to create standard polygons, such as rectangles, or arbitrary shapes. See Code Snippet 1 for an example.

#### Greater flexibility of stimulus attributes

The code syntax for changing stimulus attributes dynamically has been vastly expanded and homogenized across stimulus types, to the point that almost all attributes can be altered during runtime. The syntax for doing so has been simplified. See the stimulus updates in Code Snippet 1 for an example.

#### Application localization and translation

Another addition was the ability of the PsychoPy application’s graphical user interface to support localization into different languages. The code to make this possible was largely written by author J.R.G. To date, H.S. has translated all the elements of the application into Japanese, with other localizations possible and welcome.

#### Support for Python 3

Since 2008, the Python language has undergone a substantial change from version 2 to version 3. PsychoPy now supports both Python 2 and Python 3, so that users with older Python 2 code can continue to run their studies with no further changes, whereas users that want access to the new features of Python 3 can do so. A few of the dependent libraries, notably in specialized hardware interfaces, are still not available in Python 3–compatible versions, such that a few features still require a Python 2 installation. We therefore aim to continue supporting Python 2 for the foreseeable future.

#### ioHub and hardware polling

One of the most substantial additions to the package is the *ioHub* system for asynchronous control of hardware, written by S.S. IoHub was conceived initially for the purpose of providing a unified application programming interface (API) for eyetracking hardware, so that users could use one set of functions to control and read data from any eyetracker. It comes with integrated support for trackers from SMI, SR Research, Tobii, LC Technologies, Eye Tribe, and Gazepoint.

IoHub runs as a separate process, ensuring high-rate hardware polling without disturbing the main process that handles stimulus presentation and experimental control. The system is capable of polling data and also streaming it at very high rates—for instance, capturing all the data from a 2-kHz eyetracker. IoHub can also be used for other hardware, such as keyboards, mice, Labjack boxes, Arduinos, and so forth.

IoHub is also capable of streaming data to its own unified data file, combining the data from all the devices being polled (and data from the experiment, as sent by the main PsychoPy process), all timestamped using the same clock. This is all saved in the well-established HDF5 format. These data files allow for very high-performance hierarchical storage that can be read by most analysis packages, including MATLAB, R, and Python, thus freeing the researcher from the proprietary formats of the eyetracker itself.

Another option for eyetracking measurements in Python is PyGaze (Dalmaijer et al., 2014). PyGaze is similar to ioHub, in that it provides a unified API to several eye-gaze tracking hardware systems (EyeLink and Eye Tribe, with experimental support for SMI and Tobii trackers at the time of writing). Unlike ioHub, PyGaze makes its calls from the same thread as the main experiment/stimulus-generation thread (although the processing by the eyetracker system itself is usually carried out on another core, or even on a separate, dedicated eyetracker computer). With PyGaze, users cannot as easily combine data from different devices (e.g., button box and eyetracker) timestamped on the same clock, and they must rely on the proprietary data format of the eyetracker manufacturer and associated analysis tools.

## Open science and reproducibility

The developers of PsychoPy are advocates of open science. We believe that sharing materials, data, code, and stimuli is critical to scientific progress and hope our work has supported these goals in a variety of ways. Within the project itself, we support open science by having provided the full source code of PsychoPy since its inception in 2002 and by maintaining standard open file formats throughout.

We also encourage open scientific practices in others. By being open ourselves, by offering active support on our forum (at https://discourse.psychopy.org), and by providing many demos and free software, we hope that we set an example to the community of how science can and should be conducted. A very strong sense of community has grown around the PsychoPy project, and we believe that this is also important in encouraging open science.

With a recent grant from the Wellcome Trust, we have added our own open-source online experiment repository for users to search and upload experiments to share with each other. The site is called Pavlovia.org and can be accessed via *Git* version control, as well as directly through the PsychoPy application. This feature is currently in beta testing for the new PsychoPy3 phase of development.

## Development workflow

The workflow of PsychoPy development has changed considerably since 2008. Most notably, the version control system has moved from *SVN* to *Git*, and we have developed a test suite to ensure backward-compatibility. The source code has moved from its initial location at sourceforge.net to GitHub (https://github.com/psychopy/psychopy).

**Git:** It is now easier for users to make contributions, and for us to examine the community-contributed code. Git makes it easy to create forks and branches from the existing project, to work on them locally, and then propose changes to be folded back into the main project. The PsychoPy development repository is hosted on GitHub, which eases the workflow for contributors seeking to submit their changes and for the lead developers to review such changes.

**Tests:** Proposed code changes are now tested in an automated fashion that makes our released versions more robust to contributed errors. Using the *pytest* library, the testing now includes checks of stimulus renderings to reference images across combinations of back ends and settings, to ensure backward-compatibility. The full test suite runs automatically, checking every new contribution to the code. We believe that the combination of the extensively used test suite and the useVersion functionality yields the reliability expected for critical parts of scientific experiments.

## The growing community

We have mentioned the community aspect of the project already, but that is because it has an impact on so many aspects of the success and development of an open-source project. Open-source development works best when many people get behind a project. Without large numbers of users, there is always a danger that a project will stop being supported, due to the lack of recruitment of new developers and less impetus for the existing developers. A large community also brings the advantage of there being *many shared experiments* and teaching resources.

Figure 3 shows our growth in users, from a few hundred regular users in 2008 to a monthly-active-user count of over 21,000 in November 2018. The data are based on unique IP addresses launching the application. This systematically underestimates the actual number of users, because multiple computers on a local area network often share a single external IP address, appearing externally like a single user. Additionally, many labs disconnect their laboratory machines from the internet while running experiments, and some users choose to disable the sending of usage stats.

A small percentage of users also become contributors, in terms of providing bug fixes, new features, and documentation contributions. The project on GitHub shows an active developer community, with over 90 people contributing to the main project code. The size of these contributions naturally varies, but all fixes, even just for a typo in the documentation, are welcome. A number of contributors have devoted considerable amounts of time and effort to the project. At present, 18 contributors have each committed over 1,000 lines of updated code. In open-source software, people refer (somewhat morosely) to the Bus Factor of a project (the number of people that would have to be hit by a bus for the project to languish) and, sadly, for many projects the Bus Factor is as low as 1. The strong developer community for PsychoPy is an important ingredient in this sense; we certainly have a Bus Factor well over 1.

The third place where the community is important is in terms of mutual support. PsychoPy has a users’ forum (https://discourse.psychopy.org) based on the open-source Discourse software. This serves as a place where users ask for, and offer, support in generating experiments, where the developers discuss potential changes, and where announcements are made. The forum has just over 2,000 registered users and receives roughly ten posts per day, across a variety of categories. Users have also written a range of resources, with various workshop and online-tutorial materials, some of which have been collated at http://www.psychopy.org/resources/resources.html.

## Books

In addition to the online documentation and the user forum, there are now several books to help users learn PsychoPy. Some of the PsychoPy team have written a book teaching how to use the Builder interface (Peirce & MacAskill, 2018) and will soon release a companion book focused on programming experiments in Python. Dalmaijer (2016) uses PsychoPy to illustrate Python programming in experimental psychology. Sogo (2017) has written a textbook in Japanese on using PsychoPy to run experiments and Python to analyze data. Bertamini (2018) uses PsychoPy to teach readers how to implement a wide range of visual illusions.

## Future developments

We are now working on the next major phase of development (PsychoPy3), adding the capacity to present experiments online (and by extension, on mobile devices). In recent years, web browsers have become capable of providing access to hardware-accelerated graphics (even including GL Shader programs). This means that we can present visual stimuli in a browser with timing sufficient to synchronize to the screen refresh cycle, at least on modern hardware and software. The PsychoPy Builder interface allows this to be achieved by generating a script using HTML and JavaScript rather than the established Python code. A beta version of that system is already available, but it should be used with caution.

### Author note

*Author contributions:* All of the authors of this work have contributed their code voluntarily to the project. J.P. wrote the bulk of the code and designed the logic of both the application interfaces and the underlying Python library. He remains the primary maintainer of the code. J.R.G. contributed the next largest amount of code, most notably contributing the rating scale and the translation code, but he has really touched on nearly all aspects of the library and application, and his contribution to the project cannot be overestimated. S.S. wrote the very substantial ioHub subpackage for high-performance hardware interfacing. He also added many other features, including the TextBox for high-performance text rendering. M.M. has contributed less to the code base itself, but has been probably the most active supporter of users in the forum of anyone other than J.P. R.H. has been incredibly influential in terms of additions to the code, user support, and especially in the endeavor of improving our development and testing framework and the update to Python 3. H.S. has spent a great deal of time making sure that we appropriately support non-English users, most obviously in terms of writing a full set of translations into Japanese, but also in fixing many issues with Unicode conversions. E.K. most notably contributed and maintains the code to support switching PsychoPy versions during a script, and J.L. has provided a wide range of smaller features and bug fixes that have all very much improved the function of the software. J.P. wrote the first draft of the manuscript, but all authors were then involved in editing that draft. *Acknowledgments:* Many people have supported the project along the way, either with code contributions or by supporting users on the forum, and we are very grateful to the entire community for their work in this respect—sorry we cannot make you *all* authors! Special thanks to Yaroslav Halchenko, for providing the Neurodebian packaging and for the additional support he has provided us over the years (especially with Travis-CI testing). *Support:* The project has received small grants from the Higher Education Academy, UK, for development of teaching materials; from Cambridge Research Systems, UK, for providing support for some of their hardware (Bits#); and from the Center for Open Science, to write an interface to integrate with their server. Most recently, this work was supported by the Wellcome Trust [grant number WT 208368/Z/17/Z). *Conflicts of interest:* PsychoPy is provided completely open-source and free of charge. The authors occasionally provide consultancy in the form of training or paid support in developing experiments, although any other individuals are equally permitted to gain from providing training and consultancy on PsychoPy in this manner.
